# Comparative Study of Condensed and Hydrolysable Tannins during the Early Stages of Zebrafish Development

**DOI:** 10.3390/ijms25137063

**Published:** 2024-06-27

**Authors:** Alessandra La Pietra, Roberta Imperatore, Elena Coccia, Teresa Mobilio, Ida Ferrandino, Marina Paolucci

**Affiliations:** 1 Department of Biology, University of Naples Federico II, 80126 Naples, Italy; alessandra.lapietra@unina.it (A.L.P.); teresa.mobilio@unina.it (T.M.); 2 Department of Sciences and Technologies, University of Sannio, 82100 Benevento, Italy; rimperatore@unisannio.it (R.I.); elecoccia@unisannio.it (E.C.); paolucci@unisannio.it (M.P.)

**Keywords:** condensed tannins, hydrolysable tannins, zebrafish, embryos, metabolites

## Abstract

In this study, we present data on the effects of condensed tannins (CTs) and hydrolysable tannins (HTs), polyphenols extracted from plants, at different concentrations on zebrafish development to identify the range of concentrations with toxic effects. Zebrafish embryos were exposed to CTs and HTs at two different concentration ranges (5.0–20.0 μgL^−1^ and 5.0–20.0 mgL^−1^) for 72 h. The toxicity parameters were observed up to 72 h of treatment. The uptake of CTs and HTs by the zebrafish larvae was assessed via HPLC analysis. A qRT-PCR analysis was performed to evaluate the expressions of genes *cd63*, *zhe1*, and *klf4*, involved in the hatching process of zebrafish. CTs and HTs at 5.0, 10.0, and 20.0 μgL^−1^ were not toxic. On the contrary, at 5.0, 10.0, and 20.0 mgL^−1^, HTs induced a delay in hatching starting from 48 h of treatment, while CTs showed a delay in hatching mainly at 48 h. The analysis of gene expression showed a downregulation in the group exposed to HTs, confirming the hatching data. We believe that this study is important for defining the optimal doses of CTs and HTs to be employed in different application fields such as the chemical industry, the animal feed industry, and medical science.

## 1. Introduction

Polyphenols are a broad class of plant secondary metabolites [1,2]. Thanks to their antioxidant and anti-inflammatory activity, polyphenols are widely used in nutraceuticals and in the pharmaceutical industry [3], and as diet supplementation in functional feed [4,5,6]. As polyphenols are natural products, they are usually considered safe, but this is not always true depending on the phenolic compounds and their metabolites, the amount consumed, and the duration of exposure. Therefore, the scientific world is increasingly interested in investigating and assessing the limits between the safety and toxicity of phenolic compounds [7].

Tannins are water-soluble polyphenols that are widely used for several purposes, ranging from different industry sectors (leather, minerals, wine, and oil) to animal nutrition and biomedical uses [8]. Thanks to their ability to interact with complex proteins, they provide numerous benefits to human health, including lower risks of developing cardiovascular diseases, diabetes, cancer, and inflammation [9,10,11]. In addition, due to their antioxidant and antimicrobial activities, tannins are used as natural food preservatives to extend the shelf life of foods and to stabilize their taste, as in the case of meat, beer, and wine [12,13]. However, several studies have shown that tannins also have anti-nutritional effects due to their capability to interact negatively with food proteins or neutralize enzymes [14]. In fact, they are metal chelators, binding metal ions such as Fe^3+,^ Al^3+^, and Cu^2+^ and reducing their absorption across the gastrointestinal barrier [15].

Based on their chemical structure, tannins are classified into condensed tannins (CTs) and hydrolysable tannins (HTs). CTs, also called proanthocyanidins, are oligomers and polymers of flavonoids without a sugar core, while HTs have central core composed of a carbohydrate, mainly D-glucose, which is esterified with gallic acid or ellagic acid, forming gallotannins or ellagitannins [8,14]. Due to their ester bonds, HTs are more susceptible to hydrolysis than CTs, which give rise to the main metabolites of gallic acid or ellagic acid or other similar species [16]. Despite major differences in their chemical structures, which usually result in distinct bioactive properties, CTs and HTs often produce similar pro-oxidant or antioxidant effects, most likely depending on the concentration employed [17,18]. Moreover, Yin and collaborators showed that in vitro tannin supplementation affects embryonic development in pigs in a dose-dependent manner [19].

The zebrafish (*Danio rerio*) has become the most notable alternative animal model used for toxicological and physio-pathological studies due to its small size, rapid reproduction and development, egg transparency, and homology with higher vertebrates [20,21,22,23]. It represents an appropriate model for the screening of the bioactivity, toxicity, and side effects of plant extracts since it does not require invasive exposure procedures and provides the possibility to perform quickly reproducible dose-dependent toxicological studies by dissolving the compound directly in the growth medium [24,25,26]. Moreover, the zebrafish model also enables the possibility of evaluating off-target side effects, constituting a substantial pre-filter for the choice of the safest compound and its non-toxic dose [27].

Several studies have been carried out on the effect of natural extracts using zebrafish larvae and embryos [28,29,30]. During zebrafish development, the early stages are particularly sensitive and exposure to a plant extract can affect different tissues and organs depending on its content, the concentration used, and the period of exposure [31]. The most commonly observed alterations in embryo development are related to the hatching rate, such as delayed or premature hatching; survival; the heartbeat rate; and body malformations [31]. Interestingly, the effect of natural extracts at high concentrations on zebrafish can be assimilated to the effects of heavy-metal water contamination and pollution on physiological processes [32]. This may be due to the accumulation of these natural ingredients or their metabolites in various tissues and organs, affecting their structure and function [32].

In this context, the main objective of this study was to investigate and compare the potential toxic effects of commercial highly purified condensed tannins extracted from Quebracho wood (*Schinopsis lorentzii*) (Tan’Active QS-SOL, Silvateam S.p.a., Cuneo, Italy), and hydrolysable tannins extracted from Chinese gallnuts growing on *Rhus semialata* (Tan’Active GTC/E, Silvateam S.p.a., Cuneo, Italy), at different concentration ranges using a zebrafish embryonic model. These commercial tannins are widely used in various sectors ranging from nutraceuticals to functional food, cosmetics, animal health and nutrition, wine and beer, and many other industrial applications.

## 2. Results

### 2.1. Survival, Hatching, and Heart Rate

**Figure 1:**
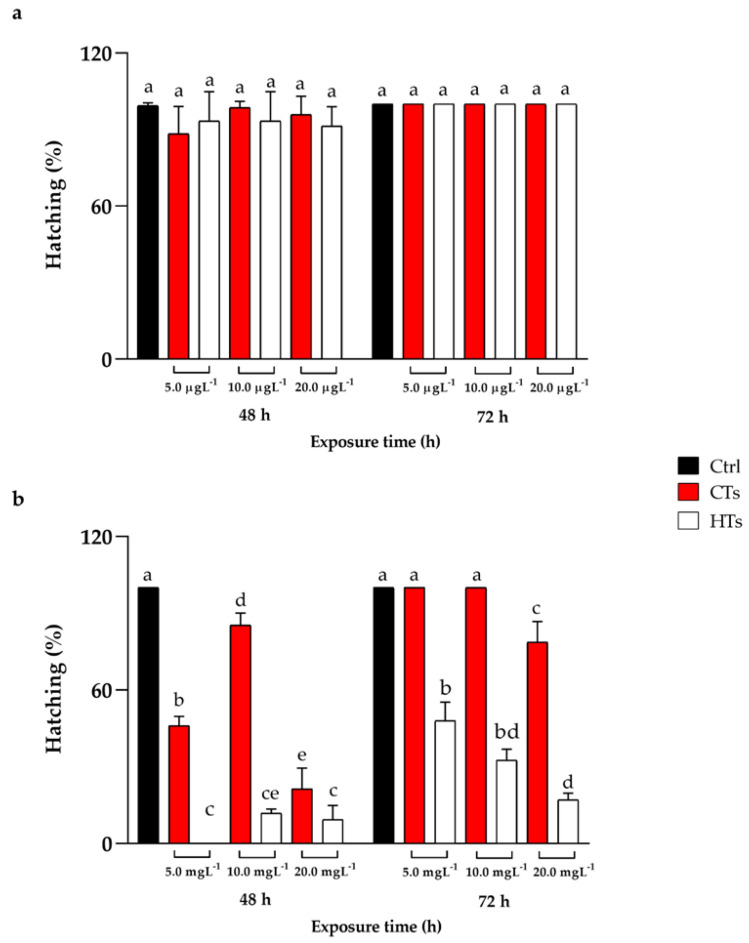
(**a**) Hatching after 48 h and 72 h of exposure to CTs and HTs at 5.0, 10.0, and 20.0 µgL^−1^. (**b**) Hatching after 48 h and 72 h of exposure to CTs and HTs at 5.0, 10.0, and 20.0 mgL^−1^. Tukey’s test: significant differences between the groups are indicated by different letters; same letters indicate no significant differences.

No significant mortality events were recorded during the treatment with CTs and HTs at all concentrations tested throughout the exposure period. The data regarding the hatching rate, reported in Figure 1a, showed that no differences emerged among the treated groups (5.0, 10.0, 20.0 µgL^−1^ of CTs and HTs) and the control group both at 48 h and 72 h of treatment.

Figure 1b shows the hatching data for the concentrations of 5.0, 10.0, and 20.0 mgL^−1^ of CTs and HTs. At 48 h of treatment, there was a delay in the hatching process for all tested concentrations of CTs compared with the control. In detail, compared to the control group, at 5.0 mgL^−1^ of CTs, the hatching rate was 46.0%, and at 10.0 mgL^−1^ of CTs, it was 85.3%, while at 20.0 mgL^−1^ of CTs, the hatching rate decreased to 21.3%. Regarding HTs, after 48 h of treatment, the hatching rate decreased at 5.0, 10.0, and 20.0 mgL^−1^ compared to the control, reaching 0%, 11.8%, and 9.33%, respectively. After 72 h of CT treatment at 5.0 and 10.0 mgL^−1^, the hatching rate was comparable to the control, while at 20.0 mgL^−1^, the hatching rate was 78.6%. With HTs at 5.0, 10.0, and 20.0 mgL^−1^, the hatching rate was 48.0%, 32.5%, and 17.1%, respectively, compared to the control.

**Figure 2:**
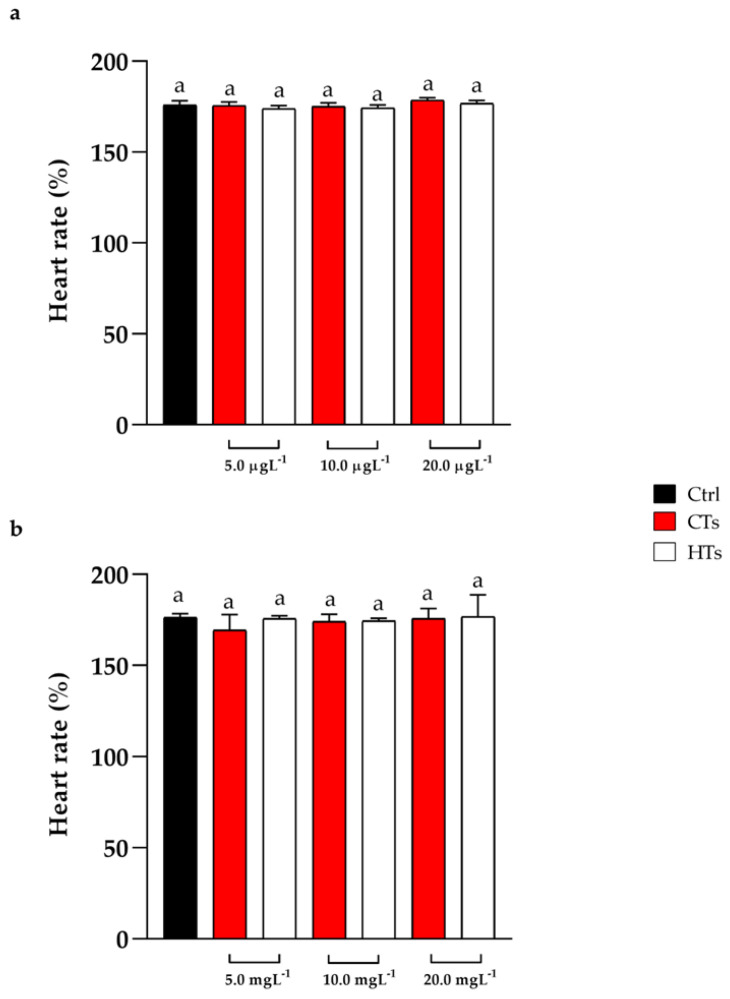
(**a**) Heart rate after 72 h of exposure to CTs and HTs at 5.0, 10.0, and 20.0 µgL^−1^. (**b**) Heart rate after 72 h of exposure to CTs and HTs at 5.0, 10.0, and 20.0 mgL^−1^. Tukey’s test: same letters indicate no significant differences.

The heart rate was recorded at 72 h of treatment. The groups exposed to CTs and HTs at 5.0, 10.0, 20.0 µgL^−1^ and 5.0, 10.0, 20.0 mgL^−1^ showed no alterations in heart rate compared with the control (Figure 2a,b).

### 2.2. Uptake of HTs and CTs

**Figure 3:**
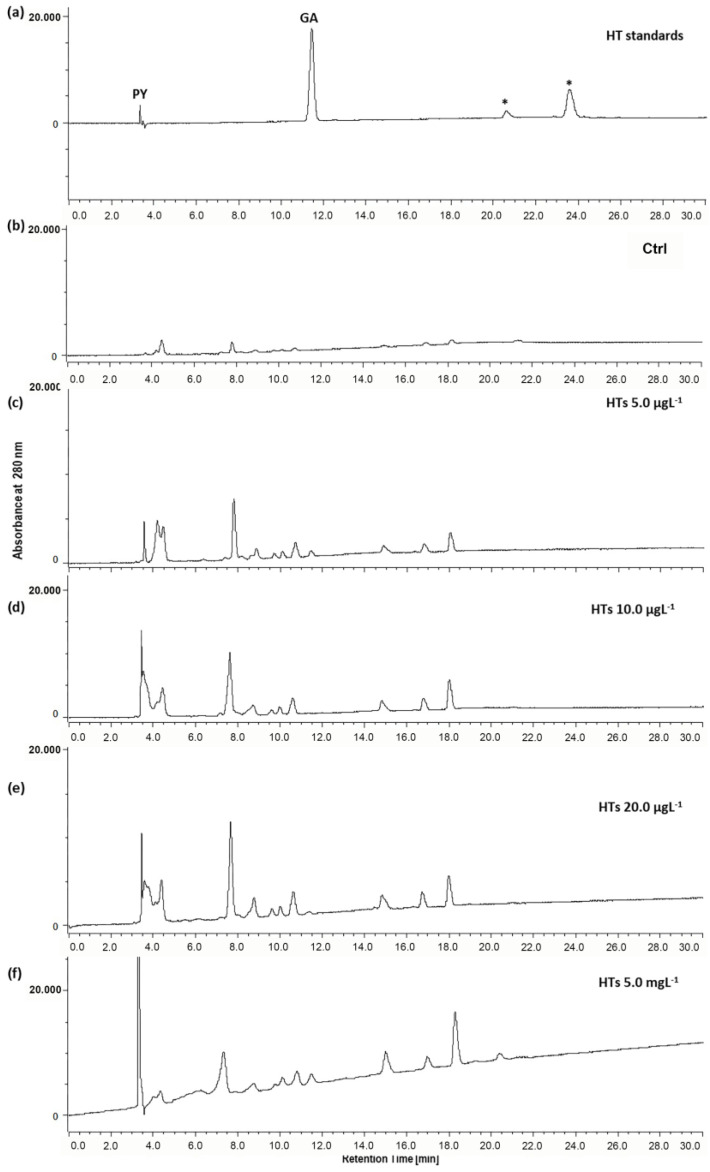
Representative HPLC profiles of (**a**) HT standards, as well as those of extracts of zebrafish larvae after 72 h of exposure to HTs at (**b**) 0.0, (**c**) 5.0, (**d**) 10.0, and (**e**) 20.0 µgL^−1^, and (**f**) 5.0 mgL^−1^. (PY, pyrogallol; GA, gallic acid; * compounds with UV–Vis spectra typical of GTs).

The uptake by the zebrafish larvae of HTs and CTs was assessed via HPLC analysis. Figure 3a shows the chromatographic profile of the HTs administered to the zebrafish larvae. The HTs used in this study were highly purified gallotannins (GTs) (Tan’Active GTC/E, Silvateam S.p.a., Cuneo, Italy); in fact, in their chromatographic profile, two peaks corresponding to the metabolites of the GTs, namely pyrogallol (PY, RT 3.5 min) and gallic acid (GA, RT 11.5 min), were identified together with two other peaks (Figure 3a, asterisks), which show a UV–Vis spectrum typical of compounds belonging to the gallotannin family [33]. The chromatographic profiles of the extracts of the larvae exposed for 72 h to the HTs (Figure 3c–f) show several peaks, among which we find those corresponding to PY and GA, which are two metabolites of HTs. These peaks are absent in the extract of unexposed larvae (Figure 3b, Ctrl).

**Figure 4:**
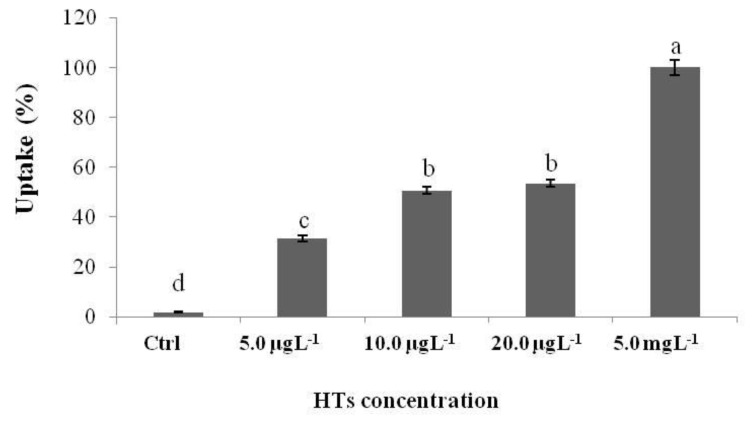
Zebrafish larvae uptake after 72 h of exposure to HTs at 5.0, 10.0, and 20.0 µgL^−1^ and at 5.0 mgL^−1^. Uptake was calculated as the percentage sum of the areas of all peaks present in the chromatogram. Data represent the means ± standard deviation (SD) of three independent experiments. Different letters indicate statistically significant differences (*p* < 0.05).

The uptake of HTs increased with the exposure concentration (Figure 4). In particular, the highest percentage of uptake was reported in the group of larvae exposed to 5.0 mgL^−1^. In the larvae exposed to 10.0 and 20.0 µgL^−1^, the uptake was similar, and the lowest uptake was reported in the larvae exposed to the lowest concentration (5.0 µgL^−1^).

Furthermore, as reported in Table 1, both the GA and PY concentrations were significantly higher in the extracts of larvae exposed to the highest concentration of HTs (5.0 mgL^−1^).

**Figure 5:**
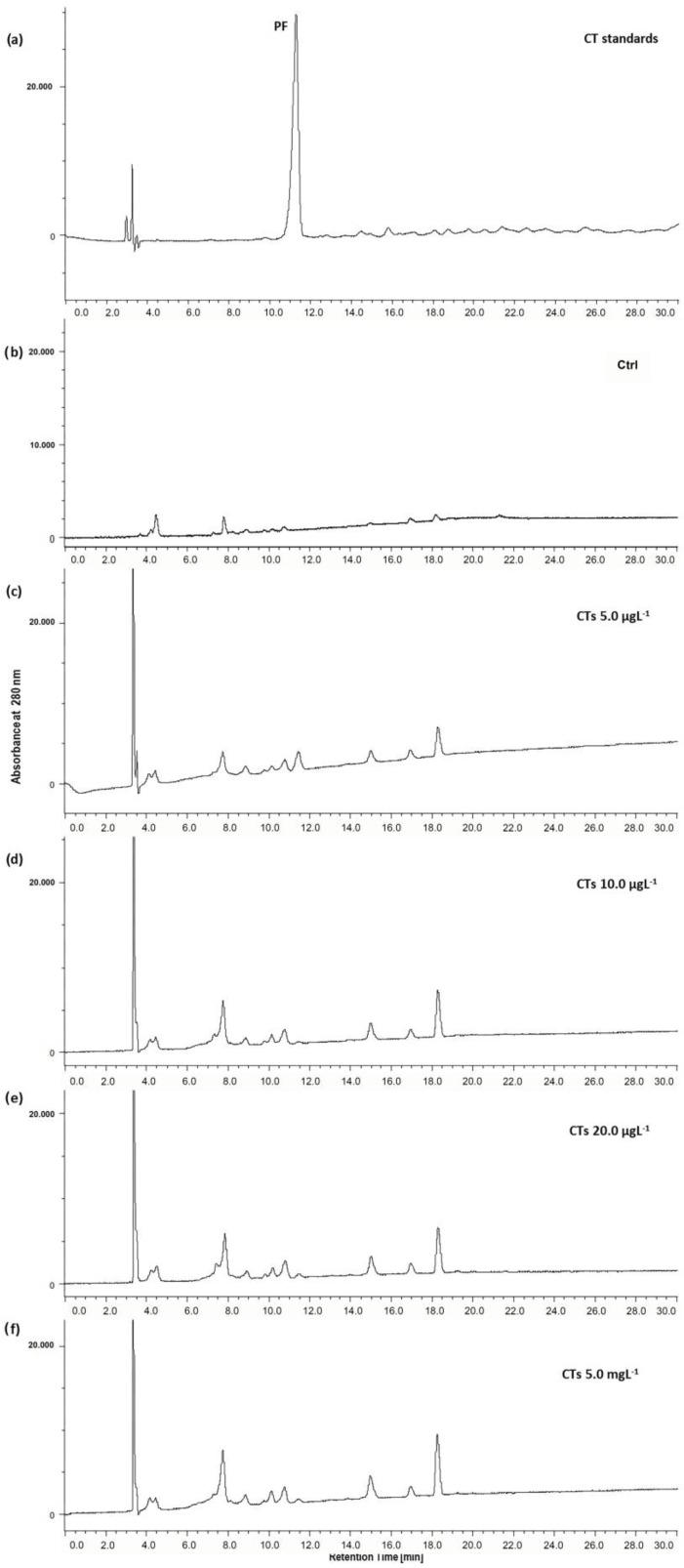
Representative HPLC profiles of (**a**) CT standards, as well as those of extracts of zebrafish larvae after 72 h of exposure to CTs at (**b**) 0.0, (**c**) 5.0, (**d**) 10.0, and (**e**) 20.0 µgL^−1^, and (**f**) 5.0 mgL^−1^. (PF, profisetinidin.).

Figure 5a shows the chromatographic profiles of the CTs administered to the zebrafish larvae.

The CTs used in this study are profisetinidin-type tannins (Tan’Active QS-146 SOL); the representative peak corresponding to profisetidin (PF, RT 11.0 min) is shown in the chromatogram. The chromatographic profiles of the extracts of the larvae exposed to the CTs for 72 h (Figure 5c–f) show several peaks, among which it is possible to identify GA and PY. These peaks are absent in the extract of unexposed larvae (Figure 5b, Ctrl).

**Figure 6:**
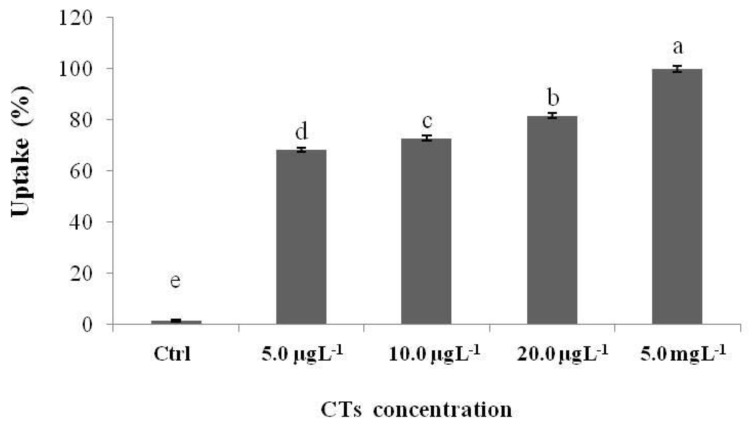
Zebrafish larvae uptake after 72 h of exposure to CTs at 5.0, 10.0, and 20.0 µgL^−1^ and 5.0 mgL^−1^. Uptake was calculated as the percentage sum of the areas of all peaks present in the chromatogram. Data represent the mean ± standard deviation (SD) of three independent experiments. Different letters indicate statistically significant differences (*p* < 0.05).

The uptake of CTs was directly proportional to the exposure concentration. In fact, the uptake percentage (Figure 6) gradually increased as the exposure concentration increased (from 5.0 µgL^−1^ to 5.0 mgL^−1^).

Furthermore, as reported in Table 2, despite the increasing concentration of exposure to CTs, the PY peak remains constant in the larval extracts, while that of GA is significantly higher in the larvae exposed to the lowest concentration of CTs (5.0 μgL^−1^).

### 2.3. Analysis of Gene Expression

**Figure 7:**
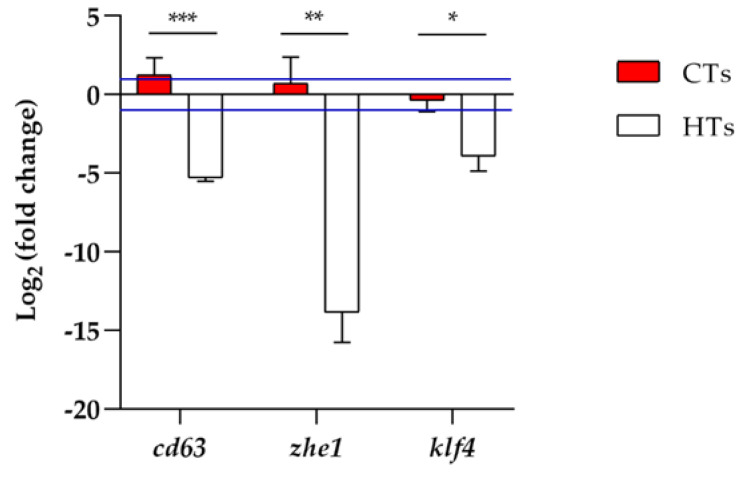
*Cd63*, *zhe1*, and *klf4* gene expression analysis results. Fold changes were calculated using the following formula: fold change = 2^−ΔΔCt^. Blue lines indicate fold change thresholds of 2 and 0.5, respectively. Values greater than 2 and lesser than 0.5 were considered significant compared to the control. The CTs vs. HTs for each gene are shown according to Student’s t-test (* *p* < 0.05; ** *p* < 0.01; *** *p* < 0.001).

A qRT-PCR analysis was conducted to evaluate the gene expression. The concentration of 5.0 mgL^−1^ was chosen because it gave a higher hatching rate at 72 h of treatment for HTs. Thus, a comparison was made between CTs and HTs at the same concentration, because 100% hatching was recorded with the CTs at 5.0 mgL^−1^. The analysis of *cd63*, *zhe1*, and *klf4* (Figure 7) showed a downregulation in the group exposed to HTs at 5.0 mgL^−1^ compared to the control group. Regarding the group exposed to CTs at 5.0 mgL^−1^, there was an upregulation only for the *cd63* gene.

## 3. Discussion

Among polyphenols, tannins have always attracted great interest due to their wide distribution in the plant kingdom and their innumerable properties, such as their antioxidant, antimicrobial, and anticancer activities and their ability to interact with proteins [18,34,35]. However, tannins and their metabolites are not free from adverse effects, and the extent of their toxic effects depends mainly on the type of tannin used and the amount consumed [14,36]. Even though both CTs and HTs have been repeatedly investigated, further research is needed to define the diversity of utilization of tannins. The data obtained in this study show that low concentrations of CTs and HTs (5.0, 10.0, and 20.0 µgL^−1^) do not cause toxicity during the early stages of zebrafish development. No mortality events or hatching/heartbeat alterations were observed. It has been reported that CTs and HTs at 5.0 µgL^−1^ prevent lipid peroxidation in rat liver mitochondria [37]. This property is related to their chemical structure due to their capacity to bind a wide range of molecules such as proteins, enzymes, and ions [38,39]. Previously published studies have shown that some polyphenols, such as tannins and their metabolites (i.e., gallic acid), counteract the toxic action of several substances or pollutants [40,41].

Our data showed that HTs at concentrations of 5.0, 10.0, and 20.0 mgL^−1^ were toxic to zebrafish embryonic development. Under normal conditions, the hatching of zebrafish embryos occurs between 48 and 72 h post fertilization (hpf) [42]. When the embryos in our study were treated with HTs, a delay in embryo hatching was observed at both 48 h and 72 h after treatment (54 and 78 hpf, respectively) for all concentrations tested. This could depend on the different metabolization or accumulation of HTs by the embryos exposed to different concentrations. In fact, the uptake of HTs increased with increasing concentrations of exposure, and it is noteworthy to observe that the greatest uptake was reported in the group exposed to the dose of HTs at 5.0 mgL^−1^ with respect to the lower doses (5.0, 10.0, and 20.0 µgL^−1^). This was reflected in the increased peak areas present in chromatographic profile of the larvae exposed to 5.0 mgL^−1^ compared those observed for the other groups, including the peaks of the two basic metabolites, GA and PY.

It is known that dietary polyphenols are metabolized to simpler bioactive molecules. The HTs used in this study were highly purified GTs. The degradation of GTs consists of a series of enzyme-catalyzed reactions that ultimately leads to the production of GA following hydrolysis of the ester bond. Then, decarboxylation would occur on GA to form PY [43].

GA is widely distributed in edible plants and possesses potent antioxidant activity and a low bioavailability due to its rapid absorption and metabolism [44]. However, higher concentrations of GA are toxic [45]. GA contains multiple hydroxyl groups, which significantly increase ROS production. Several studies suggest that high doses of GA can trigger the induction of ROS formation and subsequent apoptosis, and that ROS-induced apoptosis leads to abnormal development during embryogenesis, affecting hatching [28]. Likewise, PY promotes free radicals, leading to oxidative stress and toxicity [46].

It is probable that the synergistic effect of GA, PY, and other GT metabolites would lead to problems in hatching by interfering with the physiological processes of embryonic development. The authors of [31] indicated that exposing zebrafish embryos for 72 h to high concentrations (1–2 mgL^−1^) of natural plant extracts would results in delayed hatching or non-hatching, failure in spine development, low heart rates, delayed growth, limited movement, or death. In fact, the even-higher concentrations of 10.0 and 20.0 mgL^−1^ were not evaluated due to the embryos failing to hatch, which was probably caused by the high toxicity of these concentrations.

CTs are more widespread in nature than HTs and so are dominating the tannin market worldwide [47,48]. However, showing a different chemical structure can produce different effects with respect to HTs. Regarding CTs, the hatching rate showed a decrease mainly at 48 h of treatment, while at 72 h of treatment, only the concentration of 20.0 mgL^−1^ caused a hatching delay. Moreover, the uptake of CTs at 72 h also increased with the increasing concentration of exposure, but in a gradual manner and without major differences between the experimental groups. Likewise, the peak areas in the chromatographic profiles did not vary considerably between the experimental groups. The CTs used in this study were highly purified profisetidin tannins. Profisetinidins belong to the class of proanthocyanidins and have a powerful scavenging activity against free radicals [49]. They contain gallocatechin basic units; therefore, GA and PY are present among their metabolites. However, the concentrations of the latter are much lower than those found for HTs; therefore, they probably do not reach toxic concentrations and instead act in synergy with the PF metabolites, exerting an antioxidant effect.

Our results agree with the literature, which reports that different types of plant extract rich in polyphenols cause a hatching delay among zebrafish embryos [50,51,52].

The differences found in hatching between 48 h and 72 h of CT exposure could be explained by the change in the chorion pore size during development [53]. It could be hypothesized thataround 48 h, the chorion protects the embryo, slowing the passage of CTs [54], which could thus accumulate by clogging the pores and preventing exchanges with the external environment, leading to a delay in hatching rate across all of the concentrations tested. At 72 h, the pores change size [53], so that tannins can enter and no longer occlude the pores. In this way, development continues, and hatching occurs. Alafiatayo and colleagues reported that the reduction in hatching rate could result from an accumulation of polyphenols in the embryo subsequent to their penetration into the chorion, reaching a concentration that induces toxicity [52]. This could explain the hatching rate delay observed after 72 h of treatment with the concentration of 20.0 mgL^−1^.

The delay in the hatching process observed with the HTs at 5.0 mgL^−1^ was confirmed in the gene expression analysis at 72 h after exposure. In fact, a downregulation of the *cd63*, *zhe1*, and *klf4* genes was observed in the larvae treated with HTs. The delay in the hatching process in the presence of plant extracts has already been related to an alteration in the hatching enzyme zhe1 [29], which is involved in weakening of the chorion [55]. This hatching enzyme is a zinc-dependent metalloprotease [56], which cuts the N-terminal portions of ZP2 and ZP3, the two major glycoproteins that characterize the chorion, enabling hatching [55]. A hypothesis that would explain the slowing of the hatching process in the presence of tannins could be related to the ability of tannins to chelate metal ions [15]. In this case, CTs and HTs could retain zinc, which is necessary for the development of the hatching gland [56], delaying the hatching process. It is probable that the sequestration of zinc by HTs, and thus its unavailability, alters the upstream regulation of *zhe1*. *klf4* is a zinc-finger transcription factor, belonging to the Krüppel-like factor family, characterized by a DNA-binding domain at the C-terminal end. *klf4* regulates the differentiation of pre-polster cells, an early mesendodermal site, into hatching-gland cells, thus contributing to hatching-gland formation and its vasculature [57,58]. In addition, this gene seems to be a zinc sensor [56]. The downregulation of *klf4* confirmed the reduction in hatching in the presence of HTs, based on the zinc sequestration mechanism explained thus far. The *cd63* gene, belonging to the tetraspanin family of highly conserved transmembrane proteins, is involved in the organization of the hatching gland, and is able to regulate the position and shape of cells [59,60]. Its downregulation is in line with the delay found at the level of the hatching process, suggesting the hypothesis of an alteration in hatching-gland development. It is certain that zinc plays a key role in the normal hatching process of zebrafish [56]. A decrease in the Zn^2+^ availability could be an explanation for an alteration in the entire genetic pattern involved in the hatching process.

The differences between CTs and HTs in the gene expression analysis results are in accordance with the hatching data. In fact, in the group exposed to 5.0 mgL^−1^ of HTs, at 72 h of treatment, hatching was delayed; this is in contrast with the group treated with 5.0 mgL^−1^ of CTs, in which the hatching rate was comparable to that of the control. In the group exposed to CTs, *zhe1* and *klf4* were comparable to the control group, while *cd63* was upregulated, probably because a resumption of the hatching process occurred.

Overall, the data obtained in this study by exposing zebrafish embryos to HTs and CTs at high concentrations show how they differentially affect zebrafish development at the same concentration. It is conceivable that the different structures of these tannins affect their activity with rapidly metabolized HTs, producing PY and GA, which can act synergistically to induce toxic effects. As reported in [61], GA is readily absorbed, and this absorbed gallate induces antinutritional effects [62]. Moreover, the capability of HTs to retain zinc could further favor the delay in hatching by reducing the activity of the *zhe1* and *klf4* factors. On the contrary, CTs, having a more complex structure and a higher molecular weight with respect to HTs, show a lower bioavailability and absorbability [63], and this characteristic together with the high PF content makes CTs safer than HTs at the same concentration. However, this is true as long as the CT uptake does not reach too-high concentrations, as happened at 72 h with the concentration of 20 mgL^−1^.

## 4. Materials and Methods

### 4.1. Preparation of Solutions

We used commercial highly purified condensed tannins obtained via solvent extraction from Quebracho wood (*Schinopsis lorentzii*) (Tan’Active QS-SOL, Silvateam S.p.a., Cuneo, Italy), and hydrolysable gallotannins obtained via solvent extraction of Chinese gallnuts growing on *Rhus semialata* (Tan’Active GTC/E, Silvateam S.p.a., Cuneo, Italy). The solvents used conformed to Directive 2009/32/CE concerning extraction solvents used in the preparation of foodstuffs and ingredients; the contaminants conformed to Reg. UE 2019/934 and the International Enological Codex (OIV). We purchased Tan’Active QS-SOL with a declared purity of tannin content equal to 95%, attributable to prophysetidin-type tannins, and Tan’Active GTC/E with a declared purity of tannin content equal to 96%, attributable to highly purified gallotannins. The condensed tannins and hydrolysable tannins were dissolved in E3 medium (5 mM NaCl, 0.17 mM KCl, 0.33 mM CaCl_2_⋅2H_2_O, 0.33 mM MgSO_4_) to obtain the concentrations to be tested on embryos. The concentrations used were chosen based on those already found in the literature on *Danio rerio* and other model organisms [64,65,66].

### 4.2. Zebrafish Breeding

Eggs were obtained from 15 adult zebrafish that were housed in the Facility of the Department of Biology, University of Naples Federico II, in glass tanks, with a 14 h:10 h light/dark photoperiod, a water temperature of 28.0 °C, and a pH of 7.5. The zebrafish were fed with a commercial diet (TetraMin Tropical Flake Fish^®^, Tetra, Blacksburg, VA, USA) supplemented with *Artemia* sp. nauplii [67]. The experimental procedure obeyed National (Italian D.lgs 26/2014) and European (2010/63/EU) guidelines on the welfare of animals used for research purposes. Fertilized eggs were selected using a stereomicroscope (Leica Zoom 2000, Leica Microsystems, Wetzlar, Germany) and transferred into E3 medium.

### 4.3. Treatment of Embryos

At 6 hpf, the zebrafish embryos were exposed to 5.0, 10.0, and 20.0 µgL^−1^, as well as 5.0, 10.0, 20.0 mgL^−1^, of CTs and HTs for 72 h. A group with only E3 medium was set up as a control (Ctrl). The treatments were carried out in 6-well plates at 28.0 °C with 10 embryos per well with the addition of 10 mL of solution, which was renewed every day [68]. A total of 20 embryos were used for each group, as previously described [69], and the experiments were all triplicated according to the principle of 3Rs (Replacement, Reduction, and Refinement) [70] in order to limit the overuse of the animals.

### 4.4. Analysis of Development

Survival and hatching were followed up to 72 h of treatment (78 hpf). The number of dead embryos was determined in relation to the total number of embryos, while the number of hatched larvae was determined relative to the total number of live embryos and larvae.

The heart rate was assessed according to [71,72]. After 72 h of exposure, the larvae were placed in a hanging drop slide under a light microscope, and the heart rate was counted for 15 s and then calculated per minute.

### 4.5. Extraction of Tannins from Zebrafish Larvae

Tannins were extracted from a pool of 30 larvae with 80% (*v*/*v*) methanol. In particular, the pool of larvae was homogenized in 500 μL of 80% (*v*/*v*) methanol using a manual potter homogenizer. Then, it was vortexed for 30 s and held for 30 min at room temperature; this step was repeated three times. It was subsequently centrifuged at 13,000× *g* for 10 min and the supernatant was transferred to a sterile vial for HPLC analysis.

### 4.6. HPLC Analysis

The HPLC analysis was performed as reported in [6], using an LC-4000 Series Integrated HPLC System (JASCO, Tokyo, Japan) consisting of an oven column (model CO-2060 plus), a UV–Vis Photodiode Array Detector (model MD-2018 plus), an Intelligent Fluorescence Detector (model PF-2020 plus), a liquid chromatography pump (model PU-2089 plus), an autosampler (AS-2059 plus), and the ChromNAV software program (v. 2.0, JASCO). A C18 Luna column with a 5 μm particle size and a 25 cm × 3.00 mm I.D. was used (Phenomenex, Torrance, CA, USA).

The mobile phase consisted of 0.2% (*v*/*v*) phosphoric acid (solvent A) and 82% (*v*/*v*) acetonitrile containing 0.04% (*v*/*v*) phosphoric acid (solvent B). The temperature was maintained at 30 °C. The flow rate was 1 mL/min. The injection volume was 20 μL. The HPLC test conditions were as follows: 0–15 min, 15% B; 15–40 min, 16% B; 40–45 min, 17% B; 45–48 min, 43% B; 48–49 min, 52% B; 49–56 min, 52% B; 56–57 min, 43% B; 57–58 min, 17% B; 58–60 min, 0% B.

Peaks were detected at 280 nm and identified by comparing them with the retention times and UV–Vis spectra of the HTs and CTs, pyrogallol, gallic acid, and profisetinidin pure standards (purchased from Sigma, Milan, Italy).

The concentrations of pyrogallol and gallic acid were calculated using standard curves (Figures S1 and S2) that included five different concentrations (5, 10, 25, 50, and 100 µg/mL).

A stock solution of 1 mg/mL of each standard was prepared using methanol as the solvent. The stock solutions were then used for further dilutions.

The standard curves of pyrogallol and gallic acid were drawn by plotting the peak areas against their corresponding concentrations. The linearity was evaluated with a linear regression analysis, calculated by the least-squares regression method. The correlation coefficient, slope, and y-intercept of each calibration curve were obtained from the calibration graph.

### 4.7. Quantitative Real-Time PCR

A quantitative Real-Time PCR (qRT-PCR) analysis was carried out to analyze the expressions of genes *cd63*, *zhe1*, and *klf4* in the control group and the groups with CTs and HTs at 5.0 mgL^−1^. Total RNA from 10 zebrafish larvae per group was extracted using a Direct-zolTM RNA Miniprep Plus Kit (ZYMO RESEARCH, Irvine, CA, USA). The concentration and purity of the RNA were measured through a Nanodrop^®^ spectrophotometer 2000 (Thermo Scientific Inc., Waltham, MA, USA). Then, cDNA was synthesized from 1000 ng of the total RNA using an All-In-One 5X RT MasterMix (Applied Biological Materials, Richmond, BC, Canada). For qRT-PCR, a reaction with 2 µL of cDNA and 0.5 µL of each primer (Table S1) (Eurofins Genomics, Ebersberg, Germany) at 10.0 µM was conducted using a BlastTaqTM 2X qPCR MasterMix (Applied Biological Materials, Richmond, BC, Canada). The thermocycling conditions were as follows: 1 cycle for enzyme activation (95 °C for 3 min), 40 cycles for denaturation and annealing/extension (95 °C for 15 s, 60 °C for 1 min), and a melting-curve analysis according to the instructions provided with the StepOnePlus Real Time PCR System (Thermo Fisher Scientific Inc., Waltham, MA, USA). The expression of each gene was normalized to the *β-actin* gene and analyzed through the Ct value, using the REST software (Relative Expression Software Tool, version 1.9.12), based on Pfaffl’s method [73,74]. This experiment was conducted in triplicate.

### 4.8. Statistical Analyses

All experiments were repeated in triplicate and data were expressed as the mean ± SD. Statistical analyses were performed using GraphPad Prism Software (version 8.02 for Windows, GraphPad Software, La Jolla, CA, USA). The one-way analysis of variance (ANOVA) method followed by Tukey’s test was used to compare all experimental groups with each other. Student’s *t*-test was used to compare the expression of individual genes between the CTs and HTs. The minimum acceptable level of significance was set at *p* < 0.05.

## 5. Conclusions

This work focused on studying the comparative effect of CTs and HTs, at two different concentration ranges, on the development of zebrafish embryo. The data obtained show how two different types of tannins produce opposite effects at high concentrations, with HTs producing toxic effects compared to CTs, which are safe at the same dose. Interestingly, these effects could be due to the different absorption of CTs and HTs and to their metabolites. These findings could pave the way for a new kind of approach to study the dose-dependent effects of different tannins, in particular considering their metabolism and mechanisms of action, and lead us to find the best types and doses of tannins to use in different application fields, such as in the chemical industry, in the animal feed industry, and in medical science.

## Figures and Tables

**Table 1:** Concentration of PY and GA detected in the chromatographic profiles of the extracts of larvae exposed to different concentrations of HTs for 72 h. PY and GA concentrations are expressed as ng/larva. The concentrations of PY and GA were determined by calculating the area of their peaks in the HPLC profile and referring to standard curves. Data represent the mean ± standard deviation (SD) of three independent experiments. Values with different letters are significantly different (*p* < 0.05).

HTs	PY Concentration (ng/Larva)	GA Concentration (ng/Larva)
**Ctrl**	-	-
**5.0 μgL^−1^**	40.7 ± 3.1 ^b^	11.7 ± 1.3 ^b^
**10.0 μgL^−1^**	38.5 ± 2.9 ^b^	2.8 ± 0.7 ^c^
**20.0 μgL^−1^**	43.9 ± 2.3 ^b^	4.7 ± 0.8 ^c^
**5.0 mgL^−1^**	594.6 ± 7.3 ^a^	45.9 ± 1.2 ^a^

**Table 2:** Concentration of GA and its metabolite PY detected in the chromatographic profiles of the extracts of larvae exposed to different concentrations of CTs for 72 h. CT concentrations are expressed as ng/larva. The concentrations of PY and GA were determined by calculating the area of their peaks in the HPLC profile and referring to standard curves. Data represent the mean ± standard deviation (SD) of three independent experiments. Values with different letters are significantly different (*p* < 0.05).

CTs	PY Concentration (ng/Larva)	GA Concentration (ng/Larva)
**Ctrl**	-	-
**5.0 μgL^−1^**	386.2 ± 5.2 ^a^	16.9 ± 0.9 ^a^
**10.0 μgL^−1^**	382.7 ± 4.9 ^a^	4.3 ± 1.5 ^b^
**20.0 μgL^−1^**	383.3 ± 5.1 ^a^	7.8 ± 1.8 ^b^
**5.0 mgL^−1^**	380.3 ± 6.2 ^a^	8.3 ± 1.6 ^b^

## Data Availability

Data are available from the corresponding author upon reasonable request.

## Supplementary Materials

The following supporting information can be downloaded at https://www.mdpi.com/article/10.3390/ijms25137063/s1.
