# Supplementary material for: Comparative Study of Condensed and Hydrolysable Tannins during the Early Stages of Zebrafish Development
Source: Int J Mol Sci. 2024 Jun 27;25(13):7063. doi: 10.3390/ijms25137063 (PMC11241311; doi:10.3390/ijms25137063)
Supplement: Supplementary file 1 [file ijms-25-07063-s001.zip › ijms-3004425-supplementary.pdf]

Comparative Study of Condensed and Hydrolysable Tannins during Early Stages of Zebrafish Development

Alessandra La Pietra [1,†], Roberta Imperatore [2,†], Elena Coccia [2], Teresa Mobilio [1], Ida Ferrandino [1,*,‡] and Marina Paolucci [2,‡]

[1] Department of Biology, University of Naples Federico II, 80126 Naples, Italy; alessandra.lapietra@unina.it;teresa.mobilio@unina,it; ida.ferrandino@unina.it

[2] Department of Sciences and Technologies, University of Sannio, 82100, Benevento, Italy; rimperatore@unisannio.it; elecoccia@unisannio.it; paolucci@unisannio.it

* Correspondence: ida.ferrandino@unina.it (I.F.)

† These authors contributed equally to this work.

‡ These authors contributed equally to this work.

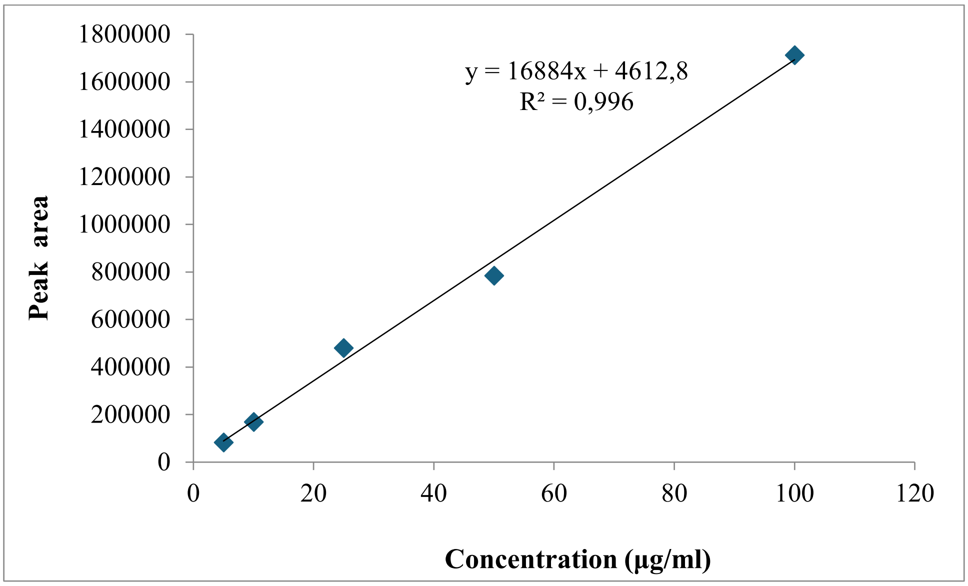

Figure S1. Standard curve of pyrogallol at concentrations of 5, 10, 25, 50 and 100 µg/ml.

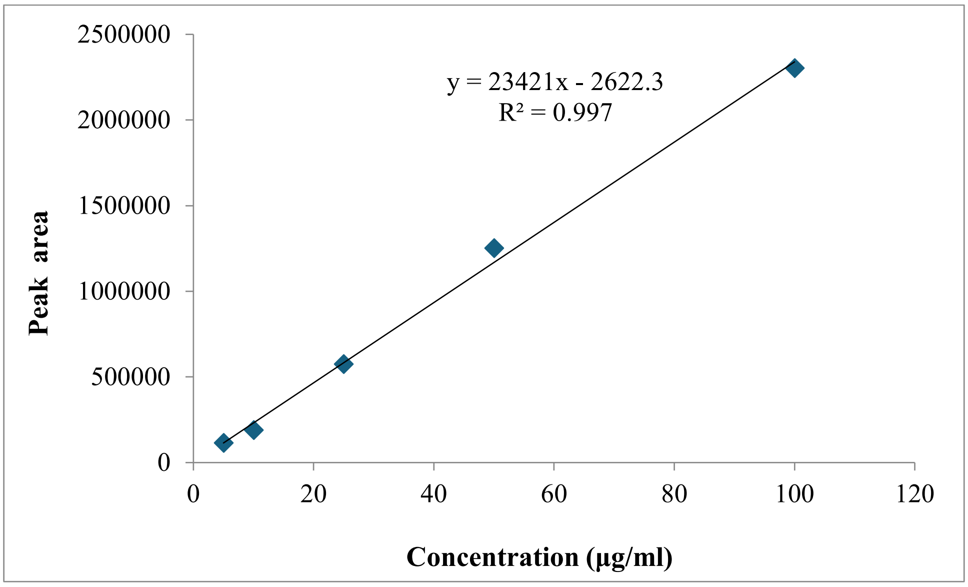

Figure S2. Standard curve of gallic acid at concentrations of 5, 10, 25, 50 and 100 µg/ml.

Table S1. Name gene, symbol, accession number, sequences, length, T_m, and references of primer used for qRT-PCR.

Name gene	Symbol	Accession number		Sequence	Length (bp)	T_m (°C)	Reference
Actin1	*β-actin*	AF057040	**F**	CGAGCAGGAGATGGGAACC	19	61.0	[1]
			**R**	CAACGGAAACGCTCATTGC	19	56.7	
Tetraspanin	*cd63*	NP_955837.1	**F**	GGAAACTCCTCTAGTGATTGGGTG	24	62.7	[2]
			**R**	CGGTGGGTTTCGTCATAGCTC	21	61.8	
Hatching enzyme 1	*zhe1*	NM_213635.2	**F**	GCCCGGTCTGGAAACCA	17	57.6	[1]
			**R**	GTCCGATCTGCACGTTTTCA	20	57.3	
Krüppel-like factor 4	*klf4*	NM_131723	**F**	TTAAGCCCAGAAGACAGCAAG	21	57.9	[3]
			**R**	GCATGTGCGCTTTCAAAT	18	51.4	

References

1. Priyam, A.; Singh, P.P.; Afonso, L.O.; Schultz, A.G. Exposure to Biogenic Phosphorus Nano-Agromaterials Promotes Early Hatching and Causes No Acute Toxicity in Zebrafish Embryos. *Environ. Sci. Nano* **2022**, *9*, 1364–1380. https://doi.org/10.1039/D1EN00659B.
2. Jung, S.; Kim, M.J.; Sellaththurai, S.; Kim, S.; Lee, S.; Lee, J. Generation of Cd63-Deficient Zebrafish to Analyze the Role of Cd63 in Viral Infection. *Fish Shellfish Immunol*. **2021**, *111*, 152–159. https://doi.org/10.1016/j.fsi.2021.01.016.
3. Vesterlund, L.; Jiao, H.; Unneberg, P.; Hovatta, O.; Kere, J. The Zebrafish Transcriptome During Early Development. *BMC Dev. Biol*. **2011**, *11*, 30. https://doi.org/10.1186/1471-213X-11-30.
